# Detecting autonomic dysfunction in patients with glaucoma using dynamic pupillometry

**DOI:** 10.1097/MD.0000000000014658

**Published:** 2019-03-15

**Authors:** Hae-Young Lopilly Park, Suk Hoon Jung, Sung-Hwan Park, Chan Kee Park

**Affiliations:** a Department of Ophthalmology and Visual Science; b Division of Rheumatology, Department of Internal Medicine, Seoul St. Mary's Hospital, College of Medicine, The Catholic University of Korea, Seoul, Republic of Korea.

**Keywords:** autonomic dysfunction, glaucoma, pupil, pupillometry

## Abstract

Autonomic dysfunction is a feature of glaucoma patients, which are reported to be related to glaucoma progression. We investigated pupil responses to a light flash using dynamic pupillometry in glaucoma patients to assess autonomic nervous system status. In total, 97 glaucoma patients, including 21 eyes of 21 glaucoma patients with cardiac autonomic dysfunction, were enrolled. Pupil reactions were assessed using 1 flash of white light after 2 minutes of dark adaptation and recorded using dynamic pupillometry. Changes in the radius of the pupil were evaluated as a function of several time-dependent and pupil/iris (P/I) diameter ratio parameters. Autonomic function was assessed using a cardiac heart-rate-variability test which performs 5 autonomic function tests and classifies patients with cardiac autonomic neuropathy (CAN). Comparison of pupil parameters between eyes with and without disc hemorrhage indicated larger P/I ratios in darkness, greater changes in the P/I ratio during examination, shorter latency to plateau, and shorter duration of constriction in eyes with disc hemorrhages. A comparison of pupil parameters between eyes with and without CAN showed larger P/I ratios in darkness, larger P/I ratios at maximum constriction, and prolonged latency to maximum constriction. The presence of CAN was significantly related to the P/I ratio in darkness and the latency of maximum constriction. Using dynamic pupillometry, we found that glaucoma patients with CAN dysfunction have larger baseline pupils in darkness and different constriction responses to light. Assessing the pupils might be a good method of identifying patients with autonomic dysfunction.

## 1 Introduction

Patients with glaucomatous damage in the normal range of intraocular pressure (IOP) has resulted in great interest in ocular blood flow in glaucoma. However, the methodology used to measure ocular blood flow remains difficult. Thus, many studies have focused on finding factors related to ocular blood flow to predict its role in the development and progression of glaucoma. Low blood pressure (BP) and nocturnal over-dipping BP are known risk factors for the development and progression of glaucoma.^[1]^ Glaucoma patients with these features show fluctuations in ocular perfusion pressure that may lead to fluctuation in ocular blood flow.^[2]^ This has been reported to originate from the extensive treatment of systemic hypertension or vascular dysregulation. Vascular dysregulation is inappropriate regulation of dilation or constriction of blood vessels, leading to cold hands, low BP, and migraines, all of which are reported risk factors for glaucoma progression, particularly in normal-tension glaucoma (NTG).^[3,4]^ The cause of vascular dysregulation is thought to be related to dysfunction in the autonomic nervous system, which regulates vessel tone, or dysfunction in the endothelial vascular layers.^[5]^

The autonomic nervous system has parasympathetic and sympathetic divisions, which control many body functions. It also affects BP control and vascular dynamics. Cardiac autonomic dysfunction has been found in glaucoma patients.^[6–10]^ We previously reported that NTG patients with cardiac autonomic dysfunction had concomitant nail fold microvascular abnormalities and higher plasma endothelin-1 levels, showing faster glaucoma progression.^[11–14]^

Thus, the assessment of autonomic dysfunction might be a tool to predict the prognosis of glaucoma and allow the customization of treatment. Several autonomic function tests are available to aid clinicians, and assessing pupil dynamics might be a simple and inexpensive approach to identifying autonomic dysfunction. The radius of the pupil is controlled by both the sympathetic and parasympathetic autonomic nervous systems in response to light.^[15]^ The pupillary radius response to an external light stimulus might provide an indirect means of assessing the integrity of neuronal pathways controlling pupil size and is used as an early indication of autonomic neuropathy in diabetic patients for the screening of autonomic dysfunction.^[16]^

In this study, we assessed the responsiveness of pupils to a light flash in glaucoma patients, examining the results according to glaucoma stage, presence of disc hemorrhage, and cardiac autonomic dysfunction (based on cardiac autonomic neuropathy [CAN] assessments). A commercially available pupilometer was used and we analyzed the data to determine dynamic pupil parameters.

## 2 Methods

### 2.1 Participants

Patients newly diagnosed with glaucoma before starting of medication were enrolled prospectively in this study. This study followed the guidelines for experimental investigations in human subjects required by the institutional review board of Seoul St. Mary's Hospital, The Catholic University of Korea, Seoul, Korea and the tenets of the Declaration of Helsinki. Written informed consent was obtained from all participants.

Each subject underwent a complete ophthalmological examination that included visual acuity, refraction, slit-lamp biomicroscopy, gonioscopy, Goldmann applanation tonometry, dilated stereoscopic examination of the optic disc, red-free fundus photography, optical coherence tomography (OCT; Cirrus OCT system, Zeiss-Humphrey Ophthalmic Systems, Dublin, CA), and a Humphrey visual field (VF) examination using the Swedish interactive threshold algorithm standard 24-2 test (Carl Zeiss Meditec, Dublin, CA).

For a glaucoma diagnosis, patients had to satisfy the following criteria: glaucomatous optic disc changes (such as diffuse or localized rim thinning, disc hemorrhage [DH], notch in the rim, or vertical cup-to-disc ratio greater than that of the other eye by more than 0.2), and glaucomatous VF loss (defined as a pattern standard deviation [PSD] [*P* < .05] or glaucoma hemifield test results [*P* < .01] outside normal limits in a consistent pattern in the Bjerrum area on both qualifying VFs), confirmed and agreed upon by 2 glaucoma specialists (HYP, CKP), best-corrected visual acuity better than 20/30, and an open angle on gonioscopic examination. Patients were excluded on the basis of any of the following criteria: who had IOP exceeding 21 mm Hg at any time during follow-up, history of any retinal disease including diabetic or hypertensive retinopathy, patients receiving any drugs affecting sympathetic or parasympathetic pupillary function, history of eye trauma or any ocular surgery, optic nerve disease other than glaucoma, history of systemic or neurological diseases that might affect the VF, any VF pattern suspicious of pathology in the optic nerve pathway other than glaucoma, and unreliable VF (defined as false negatives ≥15%, false positives ≥15%, and fixation losses ≥20%), or with nonsymmetrical pupils, misshapen pupils, or conditions affecting pupillary reflexes including diabetes mellitus. If both eyes of a patient passed inclusion and exclusion criteria, 1 eye was chosen randomly for the study.

DH at presentation, detected by stereoscopic optic disc photographs, was recorded. A DH was defined as an isolated flame-shape or splinter-like hemorrhage on the optic disc or peripapillary area, extending to the border of the optic disc.

### 2.2 Pupil examination

**Figure 1:**
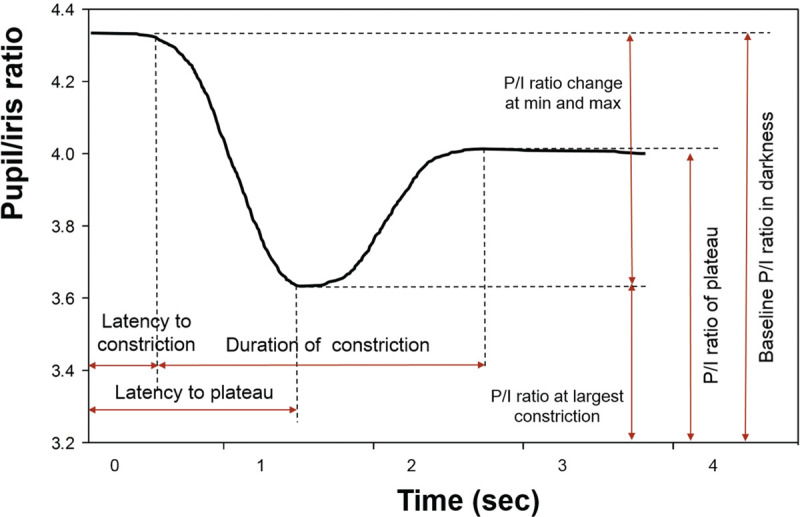
Typical pupil reflex of a healthy volunteer after a 10 ms light flash intensity of 250 cd was triggered at 0 second. The indicated parameters were defined as follows: latency to constriction (latency time to beginning of constriction), latency to plateau (latency time to reach the plateau at 75% of pre-flash pupil/iris [P/I] diameter ratio), duration of constriction, P/I diameter ratio change at min and max (change over the whole examination), P/I diameter ratio at largest constriction, P/I ratio at plateau (on reaching plateau), and baseline P/I ratio in darkness (before flash).

Pupil reactions were assessed using a flash of white light; each pupil measured twice using hand-held pupillometer (PLR-3000; Neuroptics, Irvine, CA, USA). All subjects were tested between 9 and 12 AM. For all patients, at least 8 hours of sleep was required the preceding night. The pupillometry used in this study captured the pupil response as an image frame every 1/30 seconds. A flash light with fixed intensity (250 cd) and duration (10 ms) was used. Changes in the radius of the pupil were evaluated as a function of several parameters that were time-dependent and included the ratio of pupil- and iris (P/I) diameters (P/I ratio). The pupil diameter was determined automatically for each frame in the record of the machine. Figure 1 shows how the pupil and iris diameter were determined as a pixel unit from the images obtained and then the P/I ratio was calculated.

**Figure 2:**
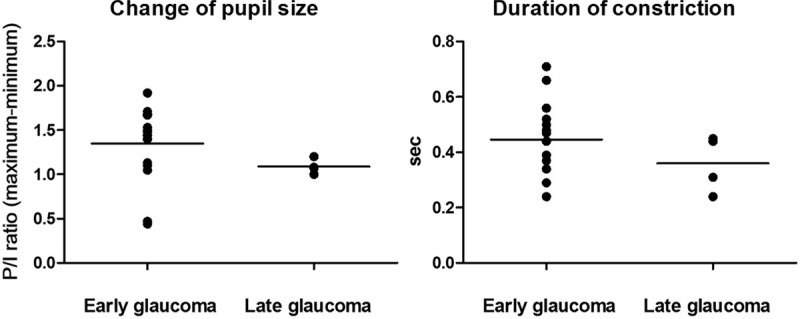
Comparison of pupil/iris diameter ratio changes at min and max and duration of constriction between early and late glaucoma.

The subject was dark adapted for 2 minutes before a single light flash was administered. The pupil response was recorded for 3 seconds. During this period, the subject was instructed to avoid or minimize blinking. During the experiments, the other eye was covered with a black fabric to avoid any external light interference. Figure 2 shows a typical pupil response from a healthy volunteer. It shows the P/I ratio in each captured frame as a function of time. The parameters of the evaluation of the pupil reflex can be defined as follows:

- Baseline P/I ratio in darkness (P/I ratio after 2 minutes of dark adaptation)
- Latency to constriction (time from flash exposure to start of constriction, when pupil diameters decreased to 90% of the pre-flash value)
- P/I ratio at largest constriction (P/I ratio when the pupil was at its smallest size)
- Latency to plateau (time from flash exposure to the smallest size of the pupil)
- During the recovery phase: P/I ratio of plateau (P/I ratio when the pupil returned to 75% of pre-flash value)
- Duration of constriction (time from when pupil diameter decreased to 90% of pre-flash value to the time when pupil returned to 75% of pre-flash value)
- P/I ratio change at min and max (baseline P/I ratio minus minimum P/I ratio after light stimulus)
- Velocity of constriction (rate of change in P/I ratio over time during the duration of constriction)

### 2.3 Assessment of heart-rate-variability

All patients were referred to the rheumatology outpatient clinic of the Department of Internal Medicine at Seoul St. Mary's Hospital, Korea. One of the coauthors (SHP) performed the physical examinations and recorded the medical status of the study participants. Hematological status, clinical chemistry, hepatitis, HIV serology, and urine analysis were evaluated to determine the health of the subject. All patients were advised to refrain from drinking caffeine or alcohol for 1 day prior to the tests. The patients were also requested to avoid activities that would affect BP, including running and jumping, for at least 2 hours prior to the test.

All of the patients were referred to the outpatient clinic of rheumatology at Seoul St Mary Hospital. They were asked whether they had a history of migraine, Raynaud phenomenon, cold extremities, orthostatic hypotension, or low BP. The assessment of heart-rate-variability was performed on a different day. This method has been described in detail elsewhere.^[7]^ Briefly, non-invasive BP was obtained from the radial artery at the wrist using an automated oscillometric device. Echocardiography was monitored for 5 minutes after 30 minutes of rest, with the study subject in the supine position. Echocardiography signals were transferred to a Medicore Heart Rate Analyzer, Model SA-3000P (Medicore, Seoul, Korea). Heart-rate recordings were carried out at rest, in deep breathing, in the Valsalva maneuver, and in active standing up. BP recordings were carried out at rest and in active standing up. The total duration of these tests was ∼30 minutes. Patients were classified as having CAN if the results of 2 or more of these 5 autonomic function tests were below age-adjusted normative values.^[7]^ The time intervals between each successive normal QRS complex were initially determined. The standard deviation of the mean of the qualified normal-to-normal intervals (SDNN) is the standard deviation of all of the normal rate-to-rate intervals in a 24-hours echocardiography recording (in ms). The group with CAN had reduced SDNN values, indicating that they had greater sympathetic activity with greater autonomic dysfunction of the heart than the group without CAN.

### 2.4 Statistical analysis

The Student *t* test was used to compare differences between groups. The Chi-Squared test was used where appropriate to compare frequencies. Linear regression analyses were used to evaluate the influence of the factors on the parameters of the pupil. The dependent variables were the baseline P/I ratio in darkness and latency to plateau. The independent variables were age, sex, diabetes mellitus (DM) diagnosis, axial length, central corneal thickness, baseline untreated IOP, VF mean deviation (MD), baseline PSD, average retinal nerve fiber layer (RNFL) thickness, presence of DH, and presence of CAN. Because DM diagnoses, presence of DH, and presence of CAN were nominal in scale, we investigated them as dummy variables, using no DM, no DH, and no CAN as the standard. A *P* value < .05 was considered to indicate statistical significance. Statistical analyses were performed using SPSS software (SPSS Inc., Chicago, IL, USA).

## 3 Results

In total, 103 eyes from 103 patients with glaucoma met the inclusion and exclusion criteria. Among them, 6 (6.7%) eyes were excluded because the pupil was covered by the upper lid and the evaluation of the exact diameter of the pupil was difficult. The remaining 97 eyes from 97 patients with glaucoma were analyzed.

Among them, 57 eyes had early glaucoma, according to the criterion MD < −6.0 dB, and 40 eyes had late glaucoma, according to the criterion MD ≥ −6.0 dB, in the VF. The late glaucoma group were older (*P* = .009) and had worse MD (*P* < .001) and PSD (*P* < .001) in the VF and thinner RNFL thickness (*P* < .001) than the early glaucoma group (Table 1). A comparison of pupil parameters between early and late glaucoma showed that the change in the P/I ratio at min and max was significantly smaller in the late glaucoma group (1.05 ± 0.11) than in the early glaucoma group (1.35 ± 0.44, *P* = .034). The duration of the constriction (0.33 ± 0.10 seconds; *P* = .020) was significantly shorter in the late glaucoma group than the early glaucoma group (0.44 ± 0.12 seconds).

**Table 1:**
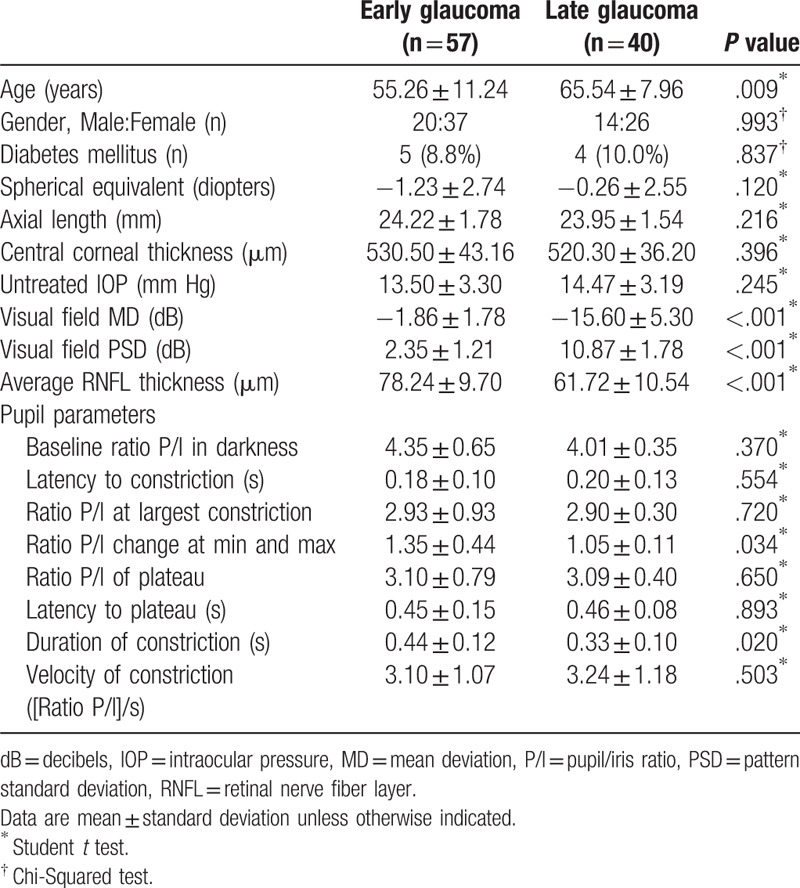
Baseline demographics and pupillometry parameters of early and late glaucoma patients.

Of the 97 eyes, 25 (25.8%) had DH at presentation. Baseline characteristics did not differ between glaucomatous eyes with DH and without DH (Table 2). Pupil examination showed that baseline P/I ratio (4.34 ± 0.53; *P* < .001) and P/I ratio change at min and max (1.30 ± 0.35; *P* < .001) were significantly greater in eyes with DH than in eyes without DH (3.10 ± 0.12 and 0.44 ± 0.12, respectively). Latency to plateau (*P* < .001) and duration of constriction (*P* = .022) were significantly shorter and the velocity of constriction (*P* = .002) was significantly faster in eyes with DH than in eyes without DH.

**Table 2:**
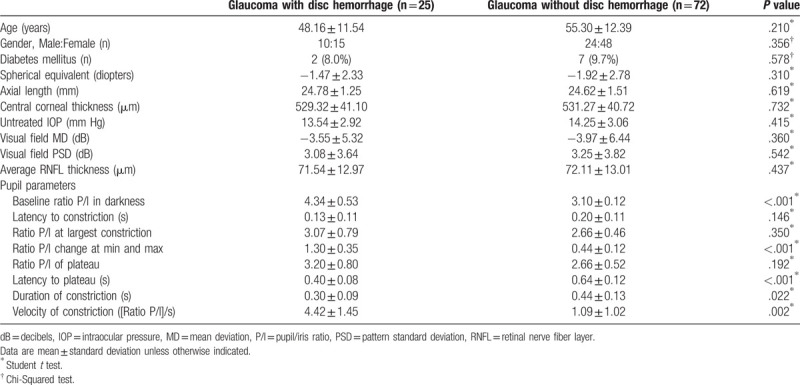
Baseline demographics and pupillometry parameters of glaucoma patients with and without disc hemorrhage.

**Figure 3:**
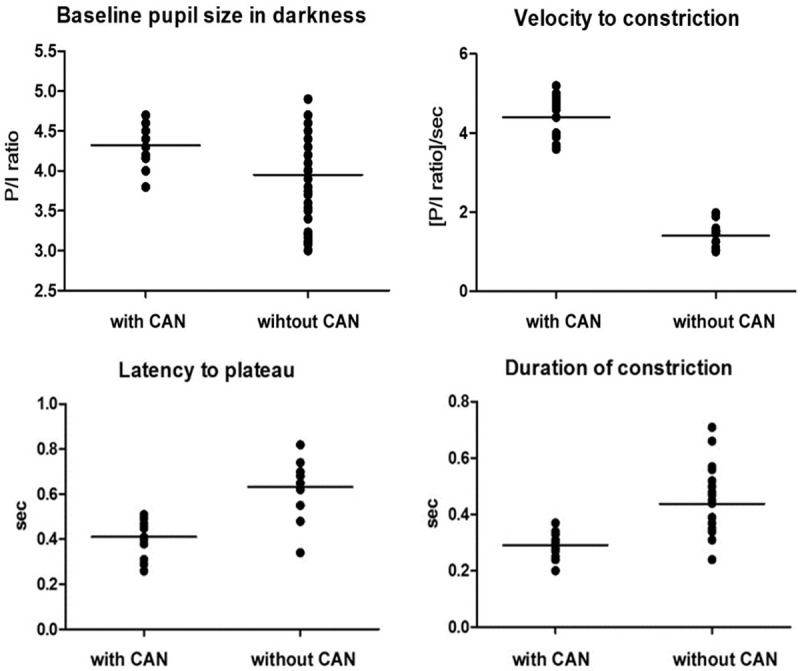
Comparison of means and standard deviations of the pupil/iris ratio at darkness, velocity to constriction, latency to plateau, and durations of constriction between glaucoma patients with and without cardiac autonomic neuropathy.

**Figure 4:**
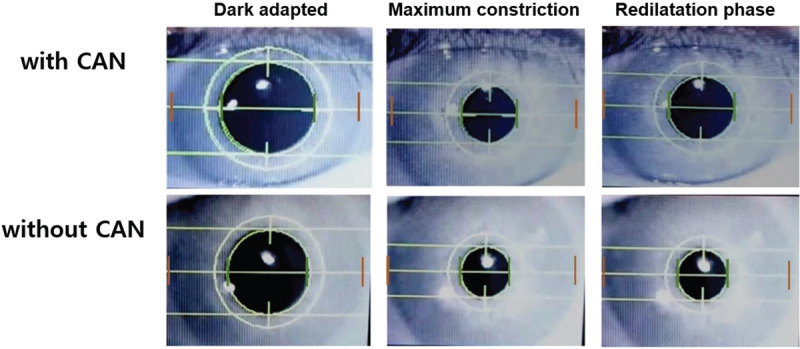
Representative cases showing difference in pupil response constriction between glaucoma patients with and without cardiac autonomic neuropathy.

Patients were further divided into those with and without CAN, based on the assessment of heart-rate-variability using the SDNN parameter. Baseline characteristics showed that glaucoma patients with CAN were significantly younger (45.75 ± 9.24 years old) than glaucoma patients without CAN (Table 3). The SDNN of the assessment of heart-rate-variability was significantly less (SDNN, 15.72 ± 3.40) in the glaucoma group with CAN than in the glaucoma group without CAN (47.54 ± 7.52; *P* < .001). Pupil examination showed that the baseline P/I ratio in darkness (4.36 ± 0.86) was significantly greater in glaucoma patients with CAN than in glaucoma patients without CAN (3.85 ± 0.80; *P* = .042). The P/I ratio at plateau was significantly larger (*P* = .049) and latency to plateau was significantly prolonged (*P* = .047*)* in glaucoma patients with CAN than in glaucoma patients without CAN. The differences in baseline P/I ratio in darkness and latency to plateau between glaucoma patients with and without CAN are shown in Figure 3. Representative images of the pupil status from glaucoma patients with and without CAN are shown in Figure 4.

**Table 3:**
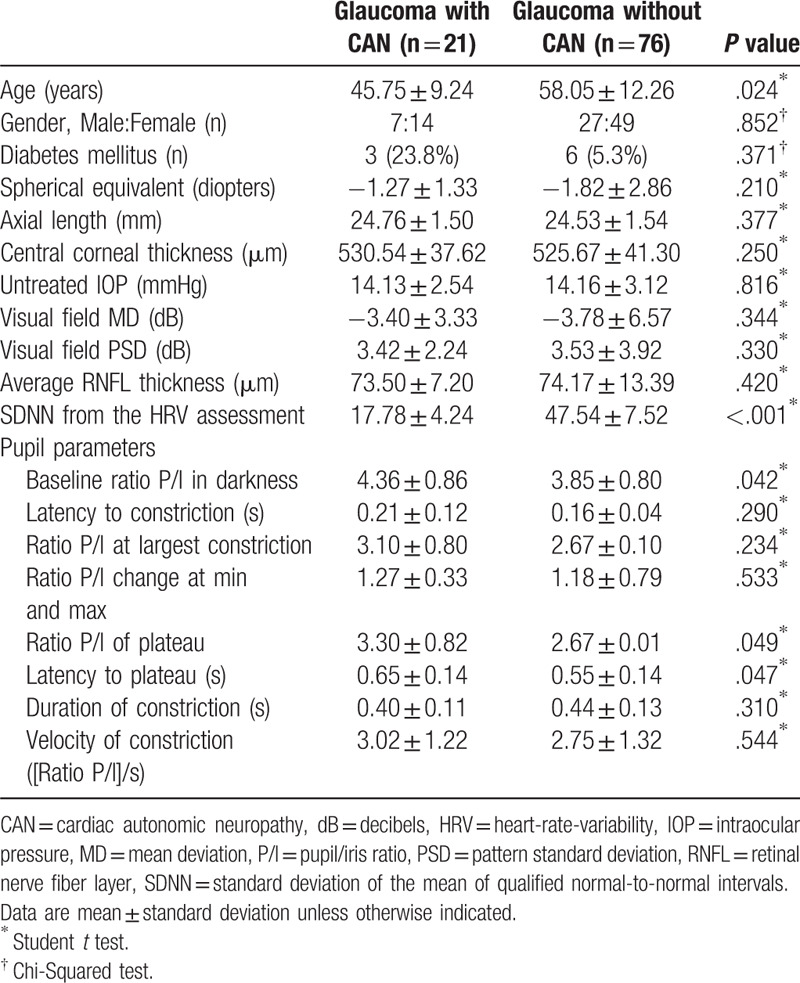
Baseline demographics and pupillometry parameters of glaucoma patients with and without cardiac autonomic neuropathy.

Table 4 shows characteristics related to baseline P/I ratio in darkness and latency to plateau in glaucoma patients. Younger age (*β* *=* −0.511; 95% confidence interval [CI] =  −0.040 to −0.015; *P* = .008) and presence of CAN (*β* *=* 0.629; 95% CI = 0.103 to 1.271; *P* = .022) were significantly associated with greater baseline P/I ratio in darkness. Worse MD of the VF (*β* *=* −0.030; 95% CI = −0.039 to −0.021; *P* < .001), thinner RNFL thickness (*β* *=* −0.017; 95% CI = −0.020 to −0.014; *P* = .007), and presence of CAN (*β* *=* 0.222; 95% CI = 0.013 to 0.433; *P* = .016) were significantly associated with latency to plateau.

**Table 4:**
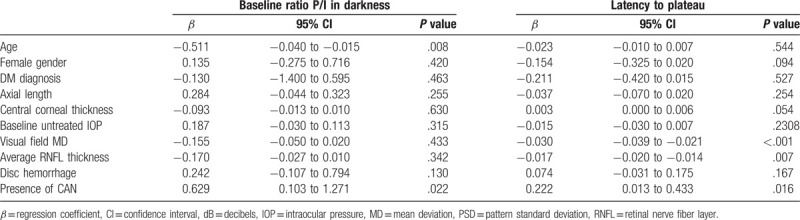
Factors associated with the pupillometry parameters rate of visual field mean deviation slope in all glaucoma patients.

## 4 Discussion

The resting size of the pupil is mainly under sympathetic control and radius reduction is a sign of diminished sympathetic outflow to the iris muscles. During the constriction phase in a light flash, pupil radius and time parameters mainly reflect parasympathetic function. Both systems are active during the recovery phase. When there is deficit in the sympathetic division, dark miosis and redilation lag are present. When there is deficit in the parasympathetic division, mydriasis is present, constriction to light do not occur, and sluggish redilation does occur. The change in pupil size in a light flash was diminished in late glaucoma patients and the duration of constriction was shorter than in early glaucoma patients. This might indicate that late glaucoma patients have parasympathetic dysfunction, affecting the pupillary reflex, or simply diminished light input, due to the advanced stage of the glaucoma. In glaucoma patients with DH, baseline pupil size was larger in darkness indicating increased sympathetic input. However, the latency to plateau and duration of constriction were shorter and the velocity of the constriction was faster, showing that the parasympathetic tone was high during the constriction phase in patients with DH. In glaucoma patients with CAN, the baseline pupil size in darkness and P/I ratio of plateau after the light flash were larger and the latency to plateau was longer, indicating that the sympathetic system was activated but not counterbalanced by the parasympathetic system. Thus, we found that glaucoma patients with DH or CAN may present autonomic dysfunction, as evaluated by an examination of the pupil.

The autonomic nervous system regulates heart rate, BP, vascular tone, and pupil response. Examining heart rate variability is a standard method of assessing systemic autonomic function. We previously reported that there is autonomic dysfunction in patients with NTG using short-term analysis of heart rate variability, and increased sympathetic activity was a distinct pattern of autonomic dysfunction.^[11]^ The increased sympathetic activity of the heart results in decreased heart-rate-variability (shown as a reduced SDNN), which is important in maintaining the ability of the heart to respond to various internal or external conditions.^[17,18]^ Systemically, increased sympathetic activity is related to dipper-type hypertension, orthostatic hypotension, and nocturnal decreases in BP, which are found in patient with NTG.^[19–21]^ It is possible that autonomic dysfunction may fundamentally contribute to various clinical presentations that indicate IOP-independent risk factors that have been reported as being associated with NTG. A 24-hours analysis of heart-rate-variability showed that there was increased sympathetic activity of the autonomic nervous system in NTG patients and that the extent of autonomic disorder correlated with the severity of glaucoma.^[9,10]^ Thus, assessing autonomic function in glaucoma patients and investigating its relationship with clinical characteristics may be important in managing these patients. Glaucoma patients with autonomic dysfunction with low heart-rate variability showed rapid central VF progression. Thus, identifying patients with these risk factors may be important in customizing treatment. Identifying changes in the autonomic nervous system in diabetic patients is important, because these patients are at risk for developing diabetic complications. The use of pupil examination in diabetic patients has been suggested to be valuable in detecting early autonomic dysfunction in high-risk groups, and it is a simple and inexpensive approach.^[16,22–24]^ We also suggest that observing pupil response could be a useful tool for identifying glaucoma patients with autonomic dysfunction, who may be at risk of disease progression. Heart rate variability is one of the standard methods to assess the systemic autonomic function, and it is a simple and noninvasive method that reflects the balance of the autonomic nervous system in regulating the heart rate.^[18,25]^ Although it is an indirect method to assess autonomic function in glaucoma patients, these patients were reported to show clinical presentations, such as migraine, DH, low BP, nocturnal hypotension, unstable mean ocular perfusion pressure, orthostatic hypotension, abnormalities in the peripheral microcirculation, and primary vascular dysregulation, which are characteristics of progressive glaucoma.^[11,12,26–32]^ Therefore, assessing pupil response in relation to CAN may be also a surrogate for identifying patients at progression risk. In addition, assessing pupil response may be simple for ophthalmologist as find out glaucoma patients with autonomic dysfunction than CAN assessment.

Interest in examining the pupil in glaucoma has grown recently. Studies in patients with asymmetric glaucoma damage between the upper and lower retina demonstrated that light stimulations projected separately to these areas produced different pupillary light reflex responses.^[33]^ Using the asymmetric involvement of glaucoma between eyes, relative afferent pupillary defects have been found to be useful in detecting and screening for glaucoma.^[34–37]^ However, the clinical application of pupil examination in glaucoma can be difficult due to variability in test duration, complexity, operator dependency, and fluctuations in pupillary responses to changes in external ambient lightening. Many investigators have tried to use computerized pupilometers or pupil perimetry in assessing glaucoma. When more objective machines, other than the swinging flashlight test are used, the specificity of glaucoma detection using the abnormal pupillary light reflex increased.^[38]^ In addition, interpreting findings from pupil examinations requires the consideration of several affecting factors. Pupil constriction speed is affected by glaucoma stage, as was found in our results, and this should be considered in interpreting pupil findings.^[39]^ Factors unrelated to optic nerve damage, such as mechanical properties of the iris, other systemic conditions, including diabetes mellitus, or use of certain medications may contribute to the variability of pupil responses. Thus, adopting pupil responses to diagnose or monitor glaucoma can be difficult. However, the use of pupil examinations to screen for autonomic dysfunction in glaucoma patients at diagnosis before starting glaucoma medication, where subjects may be at risk for glaucoma progression, may help guide the management of glaucoma. Determining the exact onset and peak contraction of the pupil is a major challenge when trying to determine whether single pupillary responses are abnormal. We showed that dynamic pupillometry can be a simple and quick technique with an objective approach to determine changes in the pupil, combined with time parameters that can also provide useful information. Among the several parameters obtained from dynamic pupillometry, the P/I ratio in darkness and the latency to plateau seem to be the ones most affected by changes in the autonomic nervous system.

Our study had several limitations. First, we had a limited sample size. Second, it is difficult to generalize our findings to all types of glaucoma or to non-Asian individuals, because our study involved mostly Korean NTG patients. NTG is the predominant glaucoma type in Korea and all the glaucoma patients included in the present study was NTG. Further study is needed to find out whether pupil responses are different among glaucoma types. Third, we wanted to investigate the relationship between the BP status of the patient and the pupillary autonomic response. However, it was difficult the elucidate significant findings related to BP parameters or the status of BP with pupillary parameters due to the limited number of patients with CAN. Finally, there are issues with the reliability and reproducibility of assessment of heart-rate-variability. In the literature, it is commonly considered a reliable measurement technique. When it is derived from stable echocardiography recorded under controlled, resting conditions, the majority of studies suggest that heart-rate-variability is a moderately to fairly reliable measurement.^[40]^ The SDNN value that we chose to classify patients has been reported to have less variability and better reproducibility than other parameters.^[40]^

In conclusion, we found that glaucoma patients with autonomic dysfunction or DH have larger baseline pupils in darkness and different pupil constriction responses. However, we should keep in mind that assessment of pupil responsiveness would not be enough to conclude for the presence of autonomic neuropathy. Assessing the pupil could be a good way to identify patients who should be further investigated for the presence of autonomic dysfunction and help guide the management of glaucoma in these patients.

## Author contributions

**Conceptualization:** Chan Kee Park, Hae-Young Lopilly Park, Sung Hwan Park, Suk Hoon Jung.

**Data curation:** Chan Kee Park, Hae-Young Lopilly Park, Sung Hwan Park, Suk Hoon Jung.

**Formal analysis:** Chan Kee Park, Hae-Young Lopilly Park, Sung Hwan Park, Suk Hoon Jung.

**Funding acquisition:** Chan Kee Park, Suk Hoon Jung.

**Investigation:** Chan Kee Park, Hae-Young Lopilly Park, Sung Hwan Park, Suk Hoon Jung.

**Methodology:** Chan Kee Park, Hae-Young Lopilly Park, Sung Hwan Park, Suk Hoon Jung.

**Project administration:** Chan Kee Park, Hae-Young Lopilly Park, Suk Hoon Jung.

**Resources:** Chan Kee Park, Hae-Young Lopilly Park, Sung Hwan Park, Suk Hoon Jung.

**Software:** Chan Kee Park.

**Supervision:** Chan Kee Park, Hae-Young Lopilly Park, Sung Hwan Park.

**Validation:** Chan Kee Park, Hae-Young Lopilly Park.

**Visualization:** Chan Kee Park, Hae-Young Lopilly Park.

**Writing – original draft:** Hae-Young Lopilly Park.

## Correction

The funding information appeared incorrectly as “This research was supported by Basic Science Research Program through the National Research Foundation of Korea (NRF) funded by the Ministry of Education (NRF-2017R1D1A1B03029229). The authors have no conflicts of interest to disclose” and has since been corrected to “The authors wish to acknowledge the financial support of the Catholic Medical Center Research Foundation made in the program year of 2015.”
